# What Prolongs a Butterfly's Life?: Trade-Offs between Dormancy, Fecundity and Body Size

**DOI:** 10.1371/journal.pone.0111955

**Published:** 2014-11-12

**Authors:** Elena Haeler, Konrad Fiedler, Andrea Grill

**Affiliations:** Department of Botany and Biodiversity Research, University of Vienna, Vienna, Austria; University of Lausanne, Switzerland

## Abstract

In butterflies, life span often increases only at the expense of fecundity. Prolonged life span, on the other hand, provides more opportunities for oviposition. Here, we studied the association between life span and summer dormancy in two closely related species of Palearctic Meadow Brown butterflies, the endemic *Maniola nurag* and the widespread *M. jurtina*, from two climatic provenances, a Mediterranean and a Central European site, and tested the relationships between longevity, body size and fecundity. We experimentally induced summer dormancy and hence prolonged the butterflies’ life in order to study the effects of such a prolonged life. We were able to modulate longevity only in Mediterranean females by rearing them under summer photoperiodic conditions (light 16 h : dark 8 h), thereby more than doubling their natural life span, to up to 246 days. Central European individuals kept their natural average live span under all treatments, as did Mediterranean individuals under autumn treatment (light 11: dark 13). Body size only had a significant effect in the smaller species, *M. nurag*, where it affected the duration of dormancy and lifetime fecundity. In the larger species, *M. jurtina*, a prolonged adult life span did, surprisingly, not convey any fecundity loss. In *M. nurag*, which generally deposited fewer eggs, extended life had a fecundity cost. We conclude that Mediterranen *M. jurtina* butterflies have an extraordinary plasticity in aging which allows them to extend life span in response to adverse environmental conditions and relieve the time limitation on egg-laying while maintaining egg production at equal levels.

## Introduction

Why some animals grow very old and others do not is still a challenge to biological science, despite massive ongoing research especially on humans [1], [2], [3], [4], [5], other vertebrates [6], [7], [8], [9], [10] and also on some invertebrate groups like *Drosophila* and butterflies [11], [12], [13], [14], [15], [16]. Of course, we know that longevity is influenced by an array of multifaceted factors, such as maternal and environmental ones, and that there are gene variants associated with increased life span. Life span differences within and among species are generally linked to differences in life-history strategies or resource availability. Any organism has to balance between the optimal investment of available resources in growth, reproduction and self-maintenance e.g., [17], [18]. As a consequence we observe different life spans, reproductive rates and body sizes as alternative strategies, frequently even within one species [19]. Generally, an extended life promises more opportunities for reproduction: longer lived females are typically more fecund e.g., in dogs [20], humans [21], butterflies [22], ants [23]. Here, we attempt to investigate intraspecific variation in life span ‘senescence plasticity’ ( = phenotypic plasticity affecting life span) within a context of its potential evolutionary benefit. Such intraspecific plasticity in live-history and concomitantly life span is advantageous when organisms have to cope with temporally heterogeneous environmental conditions [14], as herbivorous insects in temperate regions, and are obliged to synchronize their growth and reproduction with seasonal variation of the availability of hosts plants.

We therefore chose to study two Palearctic Meadow Brown butterfly species, *Maniola jurtina* and *M. nurag*, which feed on grasses as larvae and where there are pronounced intraspecific differences among females coming from Mediterranean versus Central European origin. Meadow Browns are quite ubiquitous in temperate-zone Europe, and have been studied for many aspects of their ecology in the 1970 s and 1980 s [24], [25], [26], [27], [28]. Later on, these butterflies have been neglected as it proved difficult to rear them in the laboratory, until they gained interest again due to the number of endemic species in the genus [29], [30]. *Maniola* females from Mediterranean populations, exposed to summer drought, perform a summer dormancy and live markedly longer than females that do not undergo such a developmental pause [31]. Insects often respond to the seasonal scarcity of resources with dormancy or diapause, where the latter one is a more profound and genetically programmed developmental arrest of the organism's activities and the former one denotes an inactive, but easily interruptible phase usually paralleled by delayed ovarian maturation. Dormancy may be physiologically inevitable under certain circumstances but is at the same time costly as individuals in dormancy are exposed to an extended phase of mortality risks. Moreover, they use metabolic reserves for maintenance during periods of inactivity and postpone their reproduction. All these traits are associated with potential fitness trade-offs [32]. In some insect species, this fitness trade-off is counterbalanced by increased body size of those individuals undergoing dormancy [33]. For butterflies, the role that body size plays in the relationship between dormancy, life span and fecundity has as yet been studied only scarcely in temperate regions [34], most work that has been done so far is on tropical species, e.g. [11], [35], [36], [22], [37], [38].

In butterflies, the species with the longest known life spans as adults are usually pollen or fruit feeders, e.g. the very long lived *Heliconius* butterflies [9] or the African *Bicyclus* [35], [36], [22], [37], [38]. For these tropical butterflies an extended life span may enhance their chances to produce more offspring and find adequate oviposition sites. In *Bicyclus anyana*, however, the long lived dry season morph has a lower reproductive output than the smaller wet season morph, despite its larger size and if the wet season morph is selected for longevity, females have reduced fecundity in favor of larger egg sizes. The individuals exposed to stress (e.g., temperature extremes) are often the longer lived ones in insects. In adult *Drosophila* low-temperature stress triggers a state of reproductive dormancy [39], [14] with ovarian arrest, which results in improved survival but also greater longevity of the individuals under stress. In the fly *Protophormia terraenovae*, shorter day lengths induced more cold-tolerant and longer day lengths more heat-tolerant phenotypes [40].

Our aims are to test if (1) we can experimentally induce summer dormancy in Meadow Brown butterflies that do not normally conduct a summer dormancy, (2) if the adult lifespan of the animals can be prolonged by keeping them under constant summer conditions, (3) if the two co-occurring species *M. nurag* and *M. jurtina* react concomittantly to constant summer conditions, and (4) if body-size plays a role for compensating the trade-offs for such a prolonged life?

As dormancy is usually induced by day length in temperate zone insects [41], [32], [42], [43] we exposed freshly mated female butterflies from two Mediterranean (*M. jurtina*, *M. nurag*) and one Central European populations to long-day versus short-day photoperiodic treatments.

We hypothesized that (a) Central European Meadow Brown butterflies would not conduct a dormancy even if kept under constant summer conditions, (b) Mediterranean individuals would be strongly selected for dormancy, and thus always perform a dormancy, even if environmental conditions do not so dictate, (c) *M. nurag* as a Mediterranean endemic would be adapted more strictly to drought conditions than the widespread *M. jurtina*, whereas (d) prolonged lifespan would result in decreased fecundity in both species, partly compensated by larger body size.

## Materials and Methods

### Ethics statement

The species used in this study are not protected by European, International, Italian or Austrian law. The land where the individuals were collected in Sardinia is managed by the Sardinian Forestry Department and specific permission for the collection of the butterflies used in this study was obtained from the Ente Foreste della Sardegna. In Austria, individuals were collected outside national parks, where no permit is necessary for collecting our study species.

### Animals

The female Meadow Brown butterflies used in the experiment were caught in the wild upon eclosure at the beginning of their flight period from two climatically different sites: a Mediterranean and a Central European one. As representatives from a Mediterranean climate, expected to undergo an extended reproductive dormancy [31], we collected females of the endemic *M. nurag* (abbreviated “N” hereafter, 49 individuals) and the widespread *M. jurtina* (“JM”, 54 individuals) between 29.5. –12.6.2012 at different sites in the mountains of Sardinia, Italy, from the following locations: N 39.261972/E 9.412333, N 39.261528/E 9.411778, N 39.233694/E 9.39575, N 39.238972/E 9.385806. Here, both species co-occur in partial sympatry at elevations between 500 and 950 m a.s.l [29]. To contrast these insects with Meadow Browns of Central European provenance, expected to show no or much shorter summer dormancy, 34 *M. jurtina* females were captured in a lowland habitat (150 m a.s.l.) in eastern Austria (20.6. –3.7.2012; abbreviated “JA” hereafter). In Austria, butterflies fly 1–2 months later than in Sardinia, due to climatic differences between the two regions, so all captured individuals were of approximately the same age. *Maniola* species are protandric; males hatch usually a week before females and are actively searching for freshly eclosed females, with which they mate within a few hours upon emergence (A. Grill, unpublished data). Therefore, we assume that collected butterflies had most probably mated. Indeed, >90% of the butterflies in our experiments later on laid fertile eggs (see below).

### Experimental design

Butterflies were kept under controlled conditions in two climate chambers (Binder, KBW 400, E5.1) under 24 h light-dark cycles. Females were kept individually in transparent plastic containers (1 litre volume), which were closed with a gauze lid and had humid paper towels at the bottom. Sugar water (5% fructose solution) as nutrition was available *ad libitum*. Whilst temperature was the same in both settings (24°C during light, 16°C during dark), the photoperiod was set differentially to examine the effect of day length on aestivation behavior: long-day with L16:D8 and short-day with L11:D13 cycles. The long-day treatment mimicked natural conditions around Mediterranean mid-summer, while the short-day treatment mimicked autumn conditions, when Mediterranean females in the wild start egg deposition. In total we analyzed six groups (Table 1).

**Table 1:** Number of individuals per group.

Origin	Species	Group	Treatment	Individuals
Mediterranean (Sardinia)	*Maniola nurag*	N1	long-day	34
		N2	short-day	15
Mediterranean (Sardinia)	*Maniola jurtina*	JM1	long-day	38
		JM2	short-day	16
Central European Austria	*Maniola jurtina*	JA1	long-day	22
		JA2	short-day	12

### Data collection

We checked the containers daily for eggs, removed and counted them. Each female was followed until its death. Eggs were reared until larvae hatched. From the offspring of 116 out of 129 females (∼90%) larvae hatched, confirming their mating status. Eight individuals did not lay eggs, and were eliminated from the analyses. No unusual egg-laying behavior was observed in the few females whose eggs later proved to be infertile. We therefore kept these individuals in our analyses. It is unlikely that females had laid eggs before they were caught, because they were captured soon after eclosion, right at the beginning of the flight season.

As an index for body size, we measured the length of the discoidal cell on the ventral side of the hind wing after each butterfly’s death. The discoidal cell was well preserved in all individuals and could easily be measured through a stereo-microscope at 12.5-fold magnification.

Duration of dormancy was defined as the number of days from the capture of the individual to the day the butterfly laid its first egg. We defined the date of the “first egg laid” as the first day, on which an egg was deposited, of a five day period, during which an individual butterfly laid at least four eggs. A few cases of single eggs that were laid long before all other eggs were excluded this way, as these singleton eggs did not mark the onset of the true reproductive period. Individuals that laid fewer than 20 eggs in total were excluded from all analyses related to reproduction (reproductive period, number of eggs), as we considered them to be restrained by unidentified problems during handling and maintenance (e.g. premature death), so that their inclusion into fecundity analyses would increase noise rather than confer information.

### Data analyses

To test for possible associations among the measured variables we applied general linear models with Gaussian error structure, as implemented in RStudio [44]. In a first analysis, we tested the effects of the variables day length, body-size (not transformed as normally distributed), and species or population provenance on duration of dormancy and life span (log-transformed), and for a correlation between the latter two variables. Tests were split into two partitions, which we ran separately: one for Mediterranean females of the two Meadow Brown species (*M. nurag* and *M. jurtina*), and one for *M. jurtina* females of two provenances (Mediterranean and Central European). In a second analysis, in which the models were performed separately for each of the three populations, we tested the effects of the variables on reproductive period (not transformed as normally distributed), body size (not transformed) and duration of dormancy (log-transformed) on fecundity (square root transformed), and duration of dormancy and life span (log-transformed). Size was nested within each population to prevent covariation. Inspection of residuals revealed quite good model fit in all cases. Pearson’s product moment correlation was used as a measure of association between variables. A table-wide false-discovery rate correction (FDR hereafter) was applied following Benjamini and Hochberg [45] to control for multiple tests on the same data. There was no significant collinearity in our models after FDR correction.

To get insight into the animals’ oviposition behaviour over time, we calculated a curve of the relative cumulative number of eggs for each experimental group. For this purpose, cumulative egg numbers laid per female per day were converted into the fraction relative to the total fecundity of each individual, and these values were then averaged across all butterflies within the respective experimental group. We performed a regression analysis to test for a relationship between life span and fecundity.

## Results

### Dormancy induction

**Figure 1:**
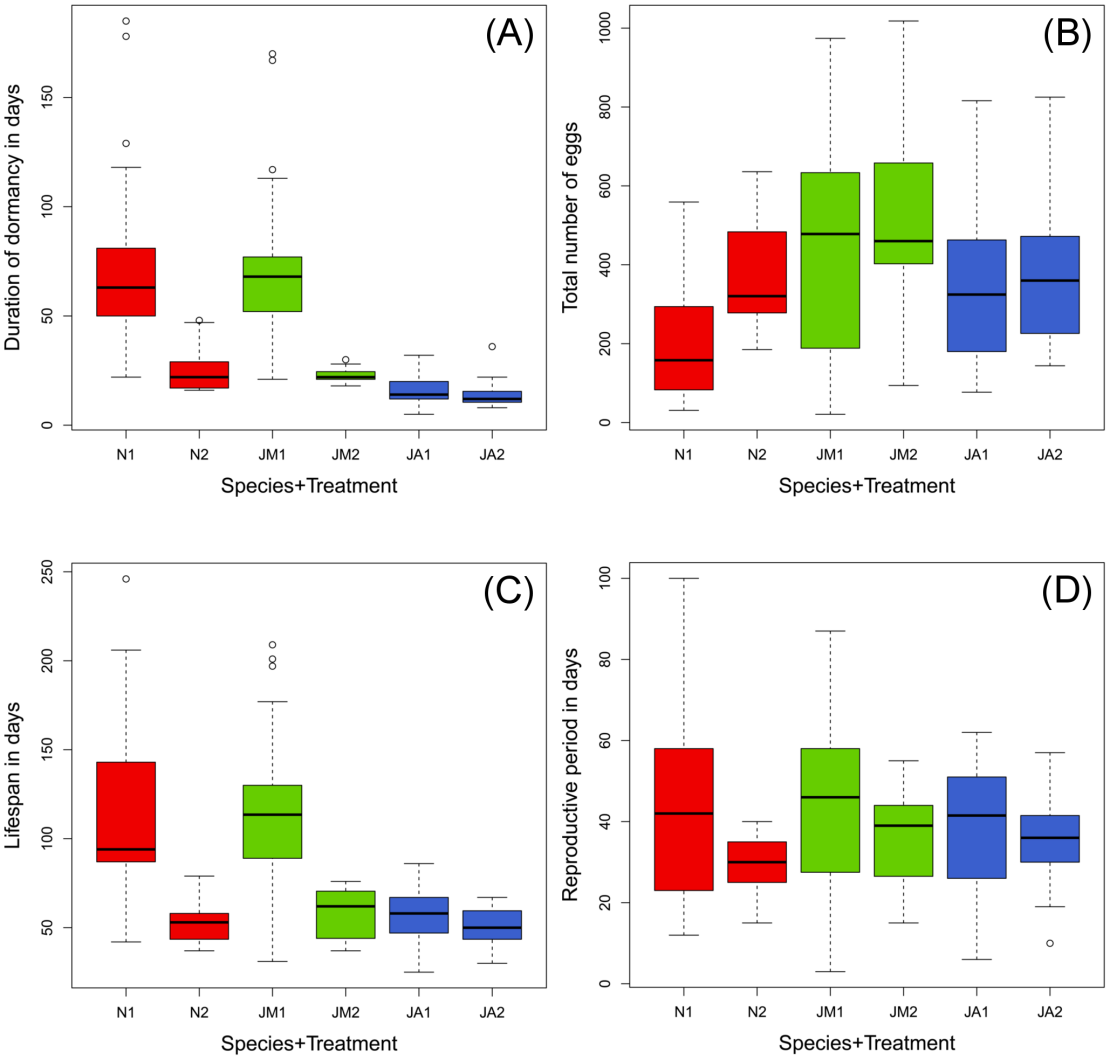
Boxplots of duration of dormancy (A), total number of eggs (B), life span (C), and reproductive period (D) across species and treatments. Long day (1) versus short day (2); red = *M. nurag* (N), green* = M. jurtina* from Sardinia (JM), blue = *M. jurtina* from Austria (JA); band = median; box = interquartile range (IQR); whiskers = lowest/highest data points within 1.5 IQR; spots = outliers.

A total of 137 individuals were reared until natural death across the two treatments; 129 of them laid eggs. Summer treatment led to delayed egg-laying (viz. reproductive dormancy) paralleled by an extremely prolonged life in Mediterranean individuals of both species (*M. nurag* and *M. jurtina*). The onset of egg-laying was delayed by up to three times (2–3 months) the time it took in the short-day treatment (3–4 weeks). In Central European individuals reproductive dormancy could not be induced (fig. 1 A).

### Comparisons between species

Both species reacted similarly to photoperiodic treatments (fig. 1 A+C, Table 2). The effect of prolonged life through the summer treatment was significant (*nurag*: t-value = 6.328, p = <0.001; *jurtina*: t-value = 6.368, p = <0.001), paralleled by prolonged duration of dormancy (*nurag*: t-value = 7.991, p = <0.001; *jurtina*: t-value = 8.187, p = <0.001).

**Table 2:** Results of linear models showing the effects of day length, body size and species or population provenance on duration of dormancy and life span.

		Dormancy		Life span	
Analysed group		*t*	*p*	*t*	*p*
Mediterranean females	**day length**	**7.991**	**<0.001**	**6.328**	**<0.001**
	species	**−2.441**	**0.017**	−1.543	0.126
	size (*jurtina*)	−0.584	0.561	0.768	0.444
	**size (** ***nurag*** **)**	**2.65**	**0.01**	**2.401**	**0.018**
	day length x species	0.154	0.878	1.088	0.279
*M. jurtina*	**day length**	**8.187**	**<0.001**	**6.326**	**<0.001**
	population	−0.971	0.335	−0.314	0.754
	size (Mediterranean)	−0.598	0.552	0.768	0.444
	size (Central Europe)	0.382	0.704	0.705	0.483
	**day length × population**	−**4.633**	**<0.001**	−**3.585**	**<0.001**

Note: Sardinian females: N = 95, *M. jurtina*: N = 86; all values marked in bold remained significant at p<0.05 after application of a table-wide false-discovery rate correction.

Total egg numbers (fig. 1 B) differed between the two species: *M. nurag* laid on average substantially fewer eggs (mean = 258, SD = 165, N = 37) than *M. jurtina* (mean = 425, SD = 247, N = 83). Mediterranean *M. jurtina* females laid more eggs (mean = 467, SD = 264, N = 50) than their Central European conspecifics (mean = 361, SD = 204, N = 33). The maximum number of eggs (1018) was deposited by a Sardinian *M. jurtina*.

### Prolonged life span in Mediterranean populations

In Mediterranean individuals, constant summer conditions on average doubled butterflies’ life span to 4 months as opposed to 2 months (fig. 1 C). There was a highly significant positive relationship between duration of dormancy and life span (*nurag*: d.f. = 35, t = 14.88, p<0.001; *jurtina*: d.f. = 48, t = 11.00, p<0.001). In contrast, Central European individuals’ life span could not be influenced by day-length and remained similar to Mediterranean individuals kept in autumn treatments, on average about 2 months (fig. 1 C).

The longest-lived individual was a *M. nurag* female that started laying eggs only after 185 days and lived for 246 days.

### Egg-laying dynamics over time

**Figure 2:**
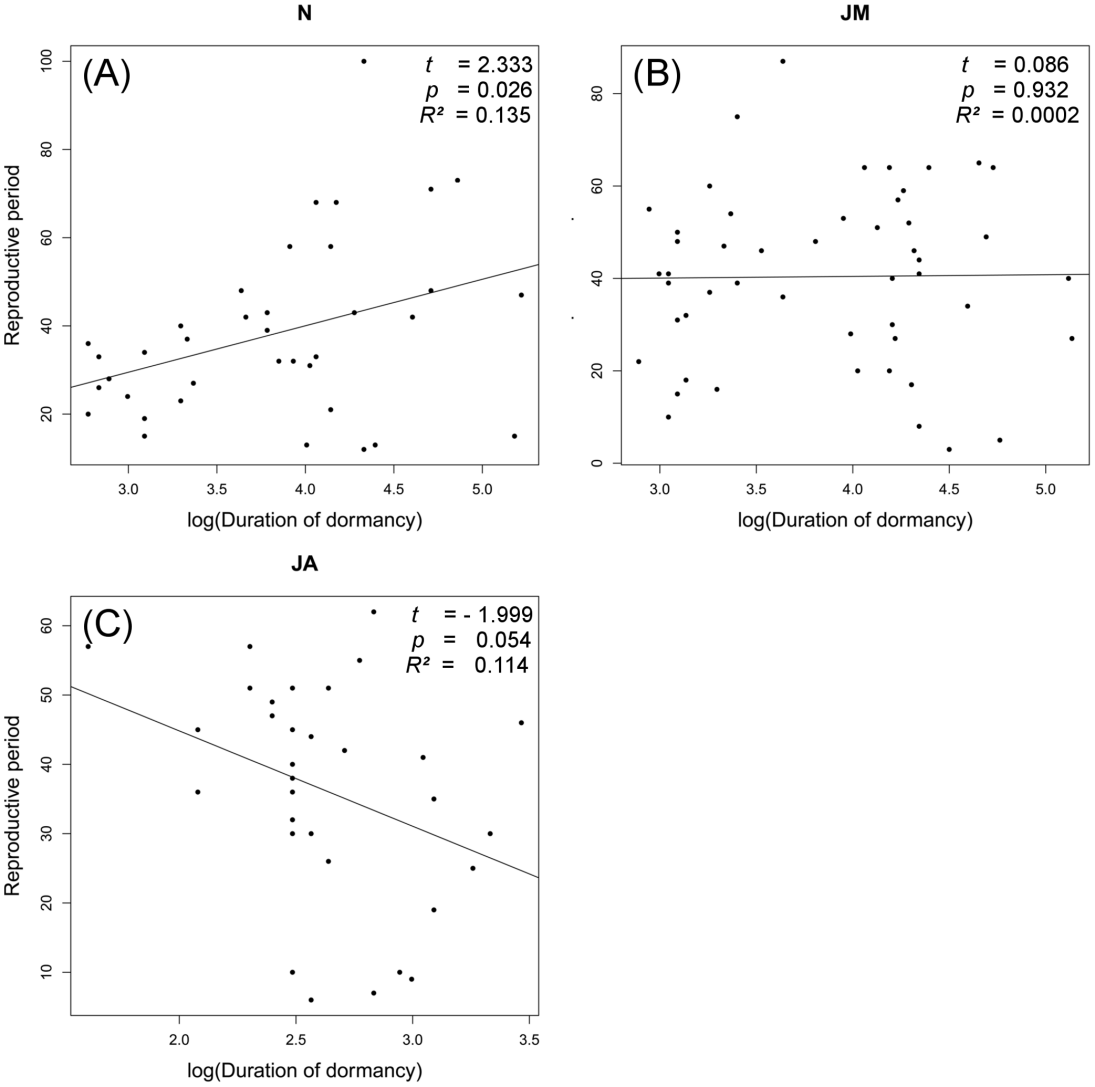
Relationships between the duration of dormancy and of the reproductive period. N = *M. nurag*, JM = *M. jurtina* from Sardinia, JA = *M. jurtina* from Austria. Results of linear regressions are shown in each plot.

Analysis of the relationship between the duration of the reproductive period and the photoperiodic treatment using the same predictor variables showed no correlations between the duration of dormancy and the duration of reproductive period (fig. 2), and each population showed a different reaction norm.

**Figure 3:**
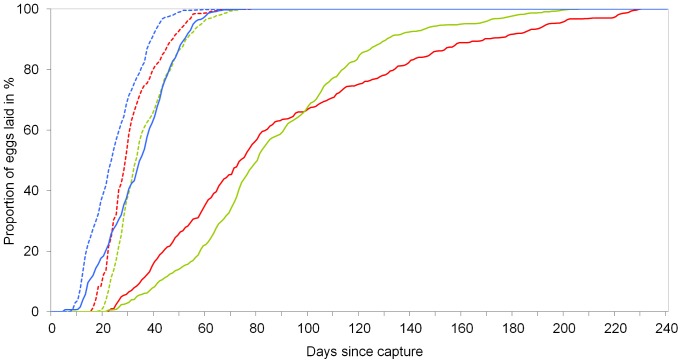
Cumulative number of eggs laid since capture for the six groups. red = *M. nurag* (N), green = *M. jurtina* from Sardinia (JM), blue = *M. jurtina* from Austria (JA); continuous lines = long-day, dotted lines = short-day.

When looking at egg-laying dynamics over time (fig. 3), autumn treatment of Mediterranean females resulted in rather quick deposition of eggs, within a few weeks after mating, similar to the behavior of Central European females in all treatments; summer treatment resulted in a delayed and much flatter oviposition curve.

### The cost of a prolonged life span: the effect of body size and species on fecundity

**Figure 4:**
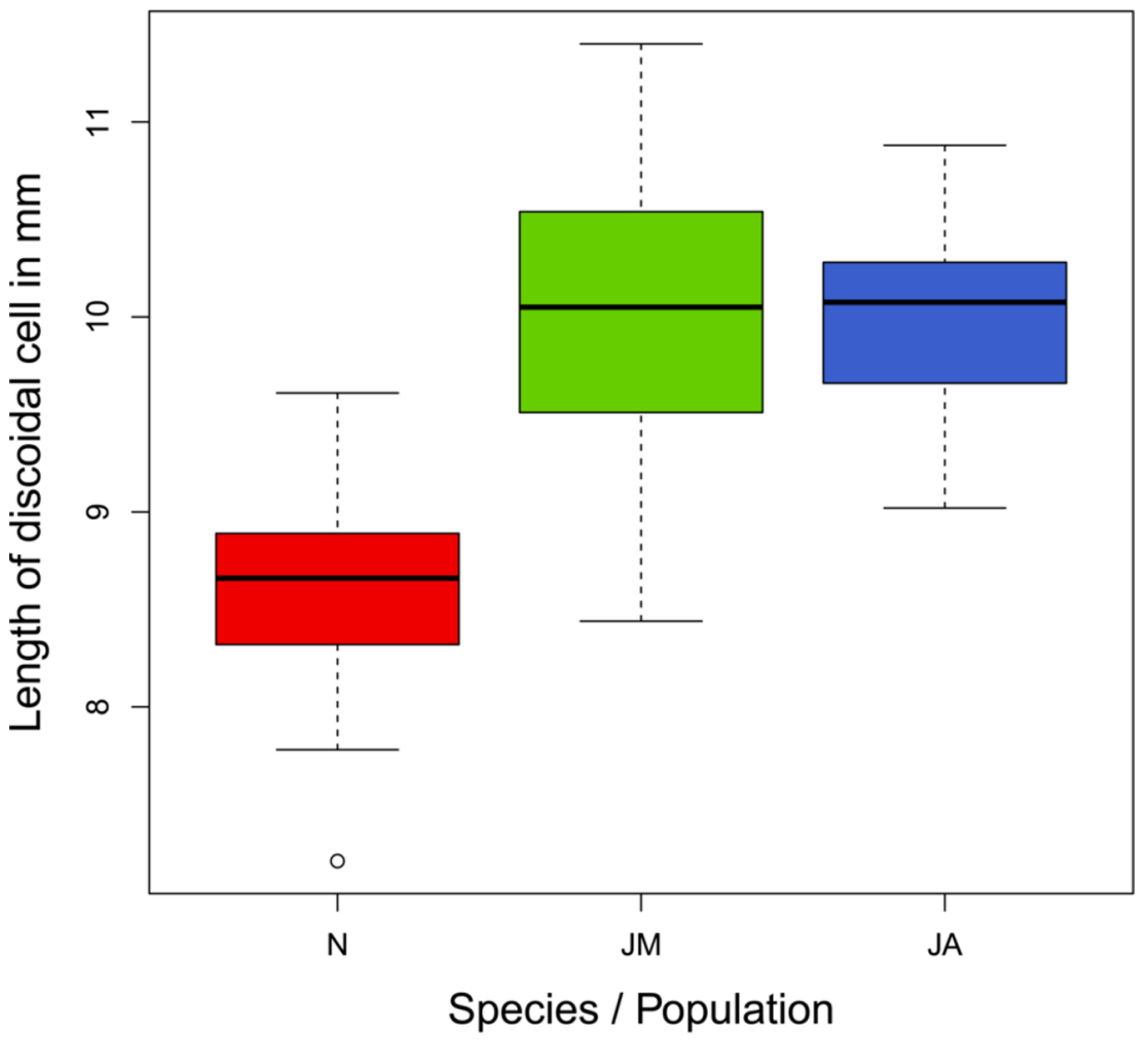
Boxplot of size of discoidal cell for each population. band = median; box = interquartile range (IQR); whiskers = lowest/highest data points within 1.5 IQR; spots = outliers.

Mean length of the discoidal cell (used as a measure for body size) was smaller in *M. nurag* with 8.6 mm (SD = 0.49, N = 49) than in *M. jurtina* with 9.9 mm (SD = 0.62, N = 88) length of the discoidal cell (fig. 4). Duration of dormancy (t-value = 2.650, p = <0.01) and life span (t-value = 2.401, p = 0.018) were positively associated with larger body size in *M. nurag*, whereas it had no effect in *M. jurtina* (Table 2).

**Figure 5:**
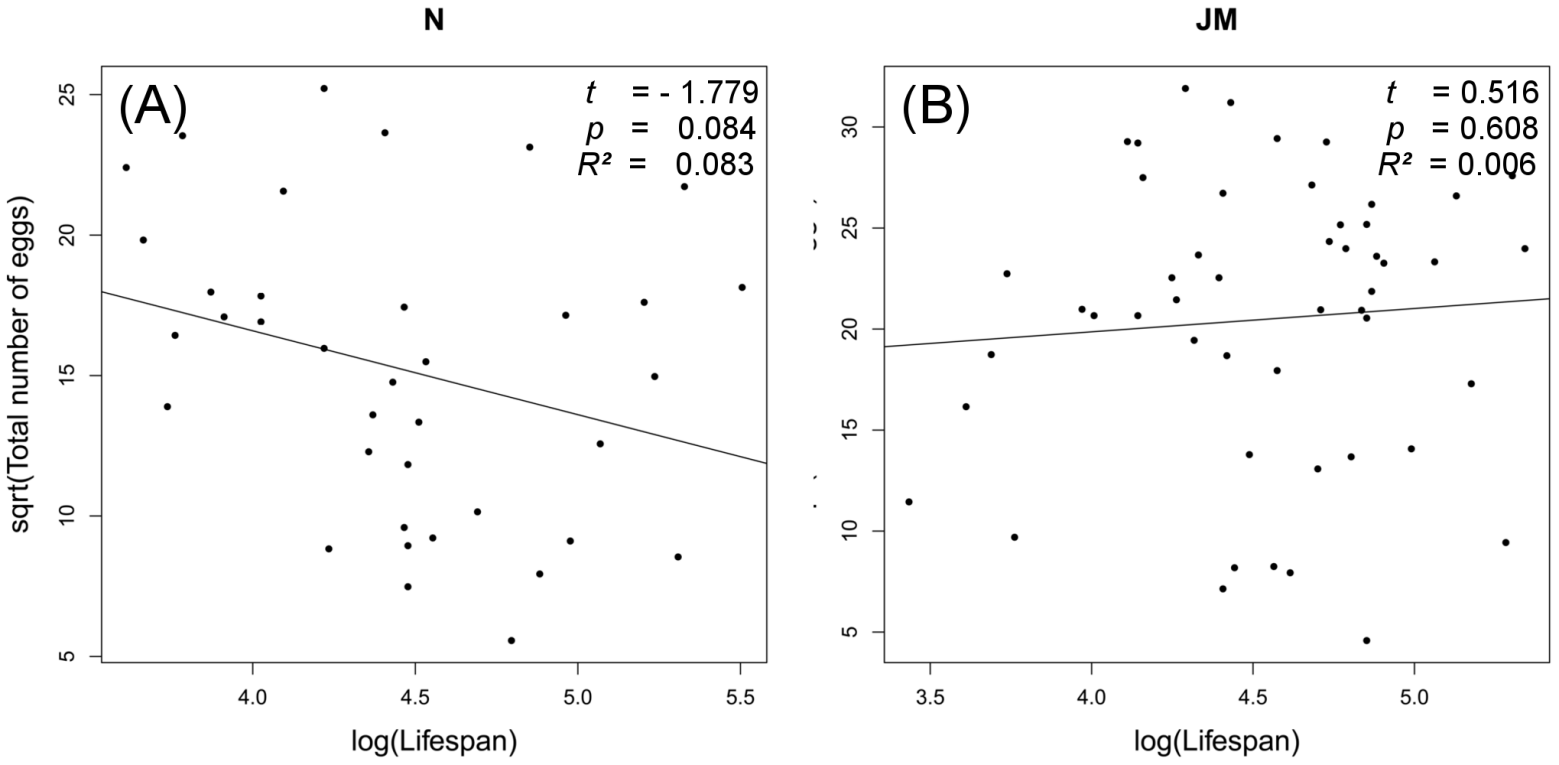
Relationship between life span and the total number of eggs in M. nurag (A) and M. jurtina (B).

Body size did not influence fecundity in any group (Table 3), but there was an implicit size-effect related to life span: in the (on average) smaller species, *M. nurag*, larger body-size had a positive effect on the length of dormancy (Table 2, and see above). Life span did not affect fecundity in either species (fig. 5) (*nurag*: t = −1.779, p = 0.084; *jurtina:* t = 0.515, p = 0.608). Length of dormancy, however, had a significant effect on egg numbers in both Mediterranean populations (*nurag*: t = −3.576, p = 0.001; *jurtina* Mediterranean = −2.781, p = 0.008). The decrease of egg numbers through extended dormancy was stronger in the species with smaller body-size, *M. nurag* (fig. 1 B). Dormancy affected fecundity (Table 3), with Mediterranean females producing more eggs (fig. 1 B). Length of the reproductive period was significantly related to fecundity in *M. jurtina* of both provenances (*jurtina* Mediterranean: t = 5.694, p = <0.001; *jurtina* Austria = 5.175, p = <0.001), but not in *M. nurag*.

**Table 3:** Results of linear models showing the effects of body size, duration of dormancy and length of reproductive period on lifetime fecundity.

Origin	Species		*t*	*p*
Mediterranean	*M. nurag*	size	1.423	0.164
		**dormancy**	−**3.576**	**0.001**
		reproductive period	2.344	0.025
Mediterranean	*M. jurtina*	size	1.974	0.054
		**dormancy**	−**2.781**	**0.008**
		**reproductive period**	**5.694**	**<0.001**
Central Europe	*M. jurtina*	size	1.944	0.062
		dormancy	−0.927	0.362
		**reproductive period**	**5.175**	**<0.001**

Note: *M. nurag*: N = 37, *M. jurtina*: Austria: N = 33, Sardinia: N = 50; all values marked in bold remained significant at *p*<0.05 after application of a table-wide false-discovery rate correction.

## Discussion

Our results support the idea that reproductive dormancy is important for the regulation of life span as has been hypothesized for the African butterfly *B. anynana* [46], [37], [38] and for a range of other organisms, as summarized in [47], but we are the first to report this from a temperate univoltine butterfly species with summer dormancy in the adult stage.

The Mediterranean Meadow Brown butterflies we studied have extraordinary plasticity to adjust their life span by means of prolonged summer dormancy in response to actual environmental conditions. Butterflies from Central European origin, which do not perform a summer dormancy in their maternal habitats, did not have this plasticity. This phenotypic plasticity obviously results from an interaction between genetic variation and the environment. The potential life span of an individual is probably determined genetically, while the effective life span depends on the conditions it meets within its lifetime, be it simply the fact that days remain long.

### The link between dormancy and longevity

Life-history theory generally predicts a negative correlation between reproductive effort and life span. Although, a longer life provides more opportunities for a butterfly to lay eggs and find suitable egg laying sites. Looking at reproductive effort as a continuous process, starting with ovarian maturation, reveals another facet of the problem. As detailed by Jervis et al. [48], the degree of egg maturation at adult emergence seems to be negatively related to life span in a phylogenetically broad array of Lepidoptera, i.e., the more eggs are ripe at eclosure the shorter is the individual’s life. Following this rationale, it is logical to conclude that Mediterranean *Maniola* have a higher potential to expand their lifespan because no eggs are ripe at eclosure.

Scali [24] described that the dormancy in *M. jurtina* is connected to a delayed maturation of the female ovaries. This explains (a) why Mediterranean females never oviposited directly after capture and (b) why Central European females oviposited distinctly earlier than the Mediterranean ones, even if both were under autumn conditions (see fig. 3). Through short-day conditions, mimicking autumn, the development of the ovaries in the (usually dormant) Mediterranean butterflies was obviously accelerated. In the summer treatment, it was delayed extremely (see fig. 1A), but finally also the females of Mediterranean provenance terminated dormancy spontaneously. This is usual for insects with artificially induced dormancy or diapause and has for example been documented in *Drosophila melanogaster* [49] where flies kept under diapause-inducing conditions suddenly resumed normal development after some time.

Adult life span of most lepidopterans typically does not exceed a few weeks [50]. So far, the longest observed active life spans in butterflies come from tropical fruit- or pollen-feeding species [51]. A classical example are *Heliconius* butterflies with life spans up to eight months in the wild [52]. Mollemann et al. [9] reported on 62 species from a tropical forest in Uganda which included twelve species that lived longer than five months and five that even lived longer than seven months; the oldest butterfly individual in the cited study, *Euphaedra medon*, endured for 293 days. Our oldest female lived for 246 days, i.e. more than eight months. To our knowledge, this is the longest life span ever recorded for a temperate-zone butterfly at the adult stage, excluding periods of inactivity during winter.

Note that during our experiments butterflies were always subject to summer temperatures, viz. as ectothermic insects they experienced metabolic rates far higher than some long-lived adult hibernators like *Gonepteryx* or *Nymphalis* species. These experimental conditions correspond, more or less, to natural conditions in their Mediterranean habitats. Summer treatment females of both *Maniola* species reached on average an adult life span of 116 days, i.e. nearly four months. Fruit feeding butterflies usually live longer than nectar feeders [51], hence it is remarkable that the life spans of the butterflies in our experiment, though nectar feeders and from temperate climates, are close to those of tropical species.

A drawback in the set-up of our experiment is, that thermoperiod automatically changed with photoperiod, so we cannot exclude that thermoperiod also affected dormancy induction and longevity. Our argument against thermoperiod, as a trigger for dormancy, is that in temperate zone insects dormancy is usually induced by day length, see also [41], [32], [42], [43]. Besides, as opposed to tropical regions, in temperate climates differences between day/night temperatures are rather unpredictable and temperature-shifts can be sudden. So, it seems unlikely that a widespread butterfly, like the Meadow Brown, would have evolved to ecologically rely on something as stochastic as the temperature of European summer nights.

### Intra- and interspecific phenotypic variation

The pronounced intraspecific differences within *M. jurtina* as a response to photoperiod indicate that there may be a genetic component to the dormancy behavior in *Maniola* spp. and consequently to the potential to double or triple life span. Under different climatic conditions, as between Mediterranean and Central European populations of *M. jurtina*, the phenotypic expression of a genotype can differ and result in larger intraspecific differences between geographically distant populations than between two species from the same location. In this respect, the data presented here confirm the results of an earlier study, where we tested the effect of amino acids added to adult diet on longevity and egg production [31] and observed that longevity and fecundity among Central European *M. jurtina* were not affected by supplementary amino acids, but only depended on the geographic provenance of the butterflies.

A similar geographic effect was reported for the large white butterfly *Pieris brassicae* [53]. Crossing experiments showed that the photoperiodic response of *P. brassicae* is heritable; when individuals with the potential for pupal summer dormancy were crossed with individuals from populations without that potential, the response in the offspring was intermediate between that of their parents. It may well be that the potential to a photoperiodically induced increase of life span is generally heritable in butterflies. However, our phylogenetic work on genetic differentiation among the species in the genus *Maniola*, conducted parallely to this study (unpublished data), as well as an earlier attempt to find evidence for genetic differentiation of the two eco-types [54] has so far only shown that the ecotypes as well as the species seem genetically almost indistinguishable.

Egg-laying strategies of Mediterranean females of both *Maniola* species in our experiment were similar (see fig. 3) and show that they are similarly adapted to the dry conditions of the Mediterranean habitat by postponing oviposition until the re-growth of larval food resources in mid/end September, after the first autumnal rainfalls. Interspecific similarities contrast with pronounced intraspecific plasticity.

A possible explanation for this could be that the physiological disposition for summer dormancy as an adult is induced by mean (or minimum/maximum) temperatures experienced as a larva during winter time. Environmental conditions during the larval stage would thus determine if an individual performs a summer diapause and switch on/off the mechanisms to do so. This would explain the similar responses of both species from the Mediterranean sites.

### Size versus fecundity

The amount of body reserves a female can convert into eggs usually increases with size [55], for Lepidoptera see [56]. This has often been shown, not only for the well-studied tropical *Bicyclus anynana*, but also for the Miami Blue butterfly *Cyclargus thomasi* [57] and temperate-zone species, for example *Leptidea sinapis* [58] and *Pararge aegeria* [34]. According to these studies, reproductive rate is supposed to decrease with female body size. Also in *Drosophila melanogaster*, larger flies generally lay more eggs [59].

In our experiment, both Meadow Brown species were able to prolong their life span through summer dormancy and, most importantly, were similarly long lived (fig. 1C). Despite differences in body size, with *M. nurag* being the smaller species, no significant negative association between fecundity and life span could be found for either species (fig. 5). But in the smaller species prolonged dormancy was paralleled by a decreased number of eggs (fig. 1B). Conclusively, in the smaller species, fecundity seems to negatively trade-off with length of dormancy while being independent of life span, while the larger *M. jurtina* was able to compensate better for the extra costs of extended dormancy and, interestingly, without fecundity loss (fig. 1B).

So far, butterflies have usually been found to react to greater longevity with reduced fecundity, like the Comma butterfly, *Polygonia c-album*, which produces two types of morphs depending on day-length and temperature experienced at the larval stage; the short-lived summer morph has a higher fecundity than the longer lived females that hibernate before reproduction [60], or also the famous *Bicyclus anynana* [61].

Selection experiments with *Bicyclus anynana* showed that the stress selected population had an increased lifespan compared to the unselected population (under benign as well as starvation conditions) and laid fewer, but larger eggs. The conclusion of the authors was that under stress induced selection females reallocate resources toward a more durable, i.e. longer lived body [37], [38]. For Nymphalid butterflies from Australia in the genus *Mycalesis*, it has been shown that the species with the stronger phenotypic variation and a more flexible breeding strategy is associated with less favorable and more unpredictable habitats [62].

Why did *Maniola* butterflies show no trade-offs between fecundity and life span? An explanation could be that Meadow Browns are able to compensate eventual resource shortcomings by increased nectar intake at the adult stage. In our experiment females had sugar water available *ad libitum*, they were thus under no starvation conditions, as opposed to the dry season form of *Bicyclus*. Also under natural conditions, Meadow Browns would be able to nectar on thistles and other flowers, even if they remain mostly inactive and cryptic within the vegetation during the hot summer.

*Maniola* butterflies exposed to summer drought seem to have found yet another solution to the trade-off between reproduction and life span, if they are large enough (i.e. from the species *M. jurtina*): they expand life spans to relieve the time limitation on egg-laying in adverse environments but maintain egg production at equal levels. They may be capable to do so, because they have more energy stored from the larval phase, than the smaller *M. nurag*.

## Conclusion

In summary, to answer our initial hypotheses our data suggests that (a) summer dormancy can be only photoperiodically induced in Mediterranean Meadow Brown butterflies, indicating that there is a genetic or developmental component to dormancy behaviour, (b) dormancy is only performed if triggered by environmental circumstances (in our experiment: photoperiod), (c) both species, *M. nurag* and *M. jurtina* react quite similarly to prolonged summer conditions, (d) but the larger species can prolong its life span without a decrease in fecundity. The latter finding is supported by the fact, that in the species *M. nurag*, larger individuals can perform longer dormancy and thus live longer.
